# Advances in Tumor Microenvironment and Immunotherapeutic Strategies for Hepatocellular Carcinoma

**DOI:** 10.32604/or.2025.063719

**Published:** 2025-08-28

**Authors:** Jiahao Xue, Jingchang Zhang, Gang Chen, Liucui Chen, Xinjun Lu

**Affiliations:** 1 Department of Biliary-Pancreatic Surgery, Sun Yat-Sen Memorial Hospital, Sun Yat-Sen University, Guangzhou, 510120, China; 2 Tianjin Key Laboratory of Acute Abdomen Disease Associated Organ Injury and ITCWM Repair, Institute of Integrative Medicine for Acute Abdominal Diseases, Tianjin Nankai Hospital, Tianjin Medical University, Tianjin, 300100, China; 3 Department of Neurology, The Second Affiliated Hospital, University of South China, Hengyang, 421001, China; 4 School of Pharmaceutical Science, Hengyang Medical School, University of South China, Hengyang, 421001, China

**Keywords:** Hepatocellular carcinoma (HCC), tumor microenvironment (TME), immunotherapy, immune checkpoint inhibitors (ICIs), combination therapy

## Abstract

Hepatocellular carcinoma (HCC) is a highly aggressive malignancy, largely driven by an immunosuppressive tumor microenvironment (TME) that facilitates tumor growth, immune escape, and resistance to therapy. Although immunotherapy—particularly immune checkpoint inhibitors (ICIs)—has transformed the therapeutic landscape by restoring T cell-mediated anti-tumor responses, their clinical benefit as monotherapy remains suboptimal. This limitation is primarily attributed to immunosuppressive components within the TME, including tumor-associated macrophages, regulatory T cells (Tregs), and myeloid-derived suppressor cells (MDSCs). To address these challenges, combination strategies have been explored, such as dual checkpoint blockade targeting programmed cell death protein 1 (PD-1), programmed death-ligand 1 (PD-L1), and cytotoxic T-lymphocyte-associated antigen 4 (CTLA-4), as well as synergistic use of ICIs with anti-angiogenic agents or TME-targeted interventions. These approaches have shown encouraging potential in enhancing immune efficacy. This review outlines the complex crosstalk between the TME and immunotherapeutic responses in HCC, emphasizing how combination regimens may overcome immune resistance. Furthermore, we discuss the remaining hurdles, including therapeutic resistance and immune-related adverse events, and propose future directions involving TME-associated biomarkers and individualized treatment strategies to improve patient outcomes.

## 1 Introduction

As of 2020, liver cancer ranks as the sixth most frequently diagnosed malignancy and stands as the third leading cause of cancer-related mortality worldwide [1]. With an estimated 500,000 new cases reported annually, its global burden continues to rise alongside increasing death rates [2]. The introduction of the Barcelona Clinic Liver Cancer (BCLC) staging system has revealed that patients with untreated hepatocellular carcinoma (HCC) have a median overall survival (mOS) of merely nine months [3]. Multiple risk factors contribute to HCC pathogenesis, including chronic infections with hepatitis B virus (HBV) or hepatitis C virus (HCV), alcohol-induced cirrhosis, nonalcoholic fatty liver disease, tobacco use, obesity, diabetes, and dietary exposures [4]. In early stages, curative interventions such as surgical resection, liver transplantation, or local ablation (e.g., radiofrequency ablation) can be effective [5]. However, recurrence remains a significant clinical challenge, with up to 70% of patients experiencing relapse within five years following curative treatment [6]. The tumor microenvironment (TME), composed of diverse non-malignant cell populations, actively contributes to HCC progression, including tumor proliferation, invasion, and metastasis. Its immunosuppressive nature not only facilitates disease progression but also impedes the success of immunotherapeutic approaches. Persistent inflammation driven by stromal and tumor-derived cytokines, chemokines, and growth factors leads to an immunosuppressive milieu, allowing tumor cells to evade immune surveillance. Complex interactions among immune cell subsets further exacerbate immune dysfunction and support carcinogenesis [7,8]. Despite the promise of systemic therapies, their effectiveness has been limited by an incomplete understanding of the TME’s regulatory mechanisms. Immunotherapy, particularly immune checkpoint blockade, offers a promising strategy by reactivating the host immune system or altering the TME. Notably, combinations such as atezolizumab plus bevacizumab (A + B) and durvalumab plus toripalimab (D + T) have shown encouraging results in clinical studies [9,10]. These regimens signify a shift toward multi-targeted strategies that simultaneously modulate different aspects of the TME, potentially enhancing treatment efficacy.

This study aims to elucidate the immunoregulatory role of the TME in HCC and to assess the therapeutic potential of combining immune checkpoint inhibitors with targeted agents. We hypothesize that mitigating immunosuppressive mechanisms within the TME through rational combination therapies can improve immune cell infiltration, reverse resistance, and enhance clinical outcomes in HCC. Ultimately, this review seeks to summarize current advancements in immunotherapy for HCC, emphasize the role of the TME in treatment response, and propose future directions for optimizing personalized therapeutic strategies.

## 2 The TME of HCC

**Figure 1:**
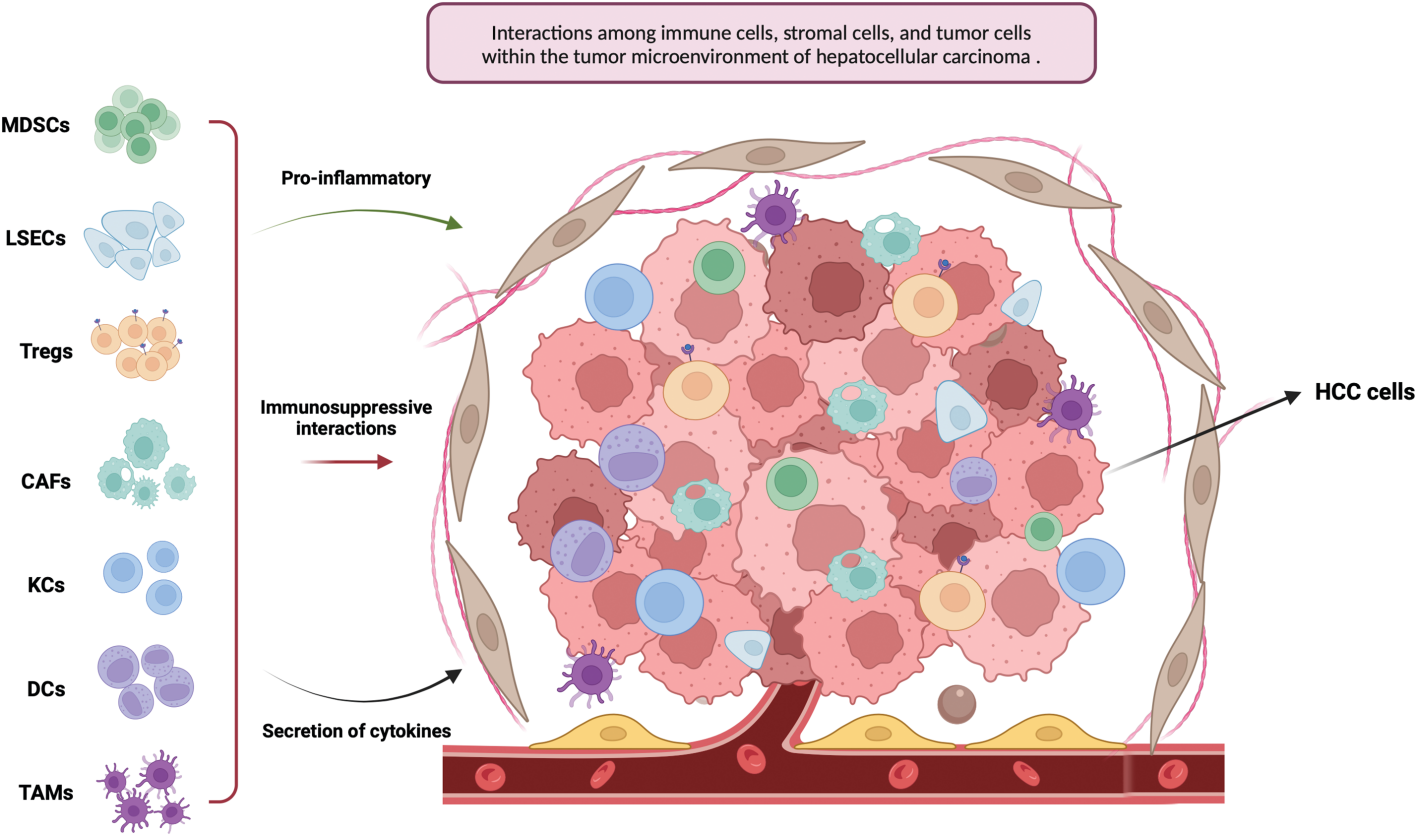
This figure illustrates the tumor microenvironment of hepatocellular carcinoma (HCC), with an emphasis on key immune and stromal cell populations, including tumor-associated macrophages (TAMs), myeloid-derived suppressor cells (MDSCs), regulatory T cells (Tregs), dendritic cells (DCs), Kupffer cells (KCs), liver sinusoidal endothelial cells (LSECs), and cancer-associated fibroblasts (CAFs) (Created in https://BioRender.com)

Emerging evidence highlights the crucial role of intercellular signaling between malignant hepatocytes and the surrounding hepatic microenvironment in driving the initiation and progression of HCC [11,12]. This complex regulatory landscape, known as the TME, influences multiple aspects of tumor biology. In HCC, the TME contributes to immune evasion by fostering immunosuppressive conditions and promoting immune tolerance, thereby facilitating tumor proliferation, invasion, and dissemination [12–14]. In this review, we examine the key elements within the TME that influence tumorigenesis, with a focus on the most relevant cellular and molecular components (Fig. 1).

### 2.1 Tumor-Associated Macrophages (TAMs)

Macrophages, originating from bone marrow–derived circulating monocytes, display notable functional plasticity. They can polarize into two major subtypes with contrasting roles: pro-inflammatory M1 macrophages and anti-inflammatory M2 macrophages, reflecting distinct activation states [15]. The M1 phenotype produces proinflammatory cytokines and reactive oxygen and nitrogen species, contributing significantly to immune activation and the elimination of tumor cells [16]. Evidence suggests that the presence of M1 macrophages within tumors correlates with improved survival in cancer patients, underscoring their tumor-suppressive role [17]. In contrast, M2 macrophages contribute to immune regulation and tissue remodeling by dampening inflammatory responses. However, this phenotype also promotes tumor progression and immune escape, and is strongly linked to poor prognosis and metastasis in HCC [18]. For instance, through C-C motif chemokine ligand 2 (CCL2) signaling, the M2 subtype can induce epithelial-mesenchymal transition (EMT), which facilitates tumor growth and invasion. This phenomenon significantly contributes to the unfavorable prognosis observed in individuals with the M2 subtype [19]. Tumor-derived Wnt ligands further promote M2 polarization, leading to immunosuppression. Therapeutic strategies targeting Wnt secretion in tumor cells or inhibiting Wnt/β-catenin signaling in TAMs have shown promise in preclinical models of liver cancer. Immunohistochemical markers such as CD86 (for M1) and CD163 or CD206 (for M2) are commonly used to differentiate macrophage subtypes in tumors. Sun et al. reported that low CD86^+^ M1 and high CD206^+^ M2 infiltration are associated with more aggressive HCC phenotypes, and the combined analysis of these markers may provide prognostic value [20]. The recent focus of research has been on populations of immunosuppressive TAMs. Specific cytokines released by HCC, such as interleukin-4 (IL-4), interleukin-13 (IL-13), colony stimulating factor 1 (CSF-1), CCL2, C-X-C motif chemokine ligand 12 (CXCL12), and connective tissue growth factor (CTGF), are known to induce TAMs derived from other sources [21]. For instance, HCC can secrete CCL2 and recruit pro-inflammatory monocyte-derived macrophages expressing C-C motif chemokine receptor 2 (CCR2) through the signaling pathway of CCL2-CCR2. A study demonstrated that these macrophages could attract regulatory T cells by producing cytokines to suppress immune responses. Additionally, TAMs and myeloid-derived suppressor cells can enhance IL-10 production, thereby inhibiting the cytotoxicity of CD8+ T cells and natural killer (NK) cells. This effect can be partially blocked by using an anti-IL-10 antibody [22]. TAMs can secrete growth factors, such as vascular endothelial growth Factor (VEGF), platelet-derived growth factor (PDGF), fibroblast growth factor (FGF), transforming growth factor-beta (TGF-β) and matrix metalloproteinases (MMPs), which can stimulate endothelial cell migration and promote neovascularization. This process is known as angiogenesis and may contribute to cancer development [23]. Research shows sorafenib can increase macrophage recruitment and VEGF levels in HCC patients. Targeting receptors or signaling proteins is crucial to impede tumor progression and macrophage properties.

### 2.2 Myeloid-Derived Suppressed Cells (MDSCs)

The MDSCs encompass a heterogeneous population of immature myeloid cells, comprising granulocytic or polymorphonuclear MDSCs (PMN-MDSCs) and monocytic MDSCs (M-MDSCs). PMN-MDSCs exhibit morphological similarities to N2-polarized neutrophils, while M-MDSCs share a resemblance with M2-polarized macrophages [24]. In HCC, MDSCs are key mediators of immune tolerance, and their accumulation within tumor tissues is strongly correlated with poor prognosis [25]. Within the TME, MDSC expansion is driven by a variety of cytokines and chemokines. Tumor-derived factors such as granulocyte colony-stimulating factor (G-CSF), granulocyte-macrophage colony-stimulating factor (GM-CSF), monocyte chemoattractant protein-1 (MCP-1), and interleukin-1 beta (IL-1β) enhance MDSC recruitment. In addition, hepatic carcinoma-associated tumor-associated fibroblasts (TAFs) promote MDSC development via the IL-6/STAT3 signaling pathway [26]. By activating the EZH2/NF-κB signaling pathway, the cell cycle-related kinase (CCRK) protein found within hepatomas can cause an accumulation of MDSCs. The studies have demonstrated that MDSCs are attracted to VEGF and can enhance its availability through the production of MMP9. Additionally, MDSCs possess the ability to influence other cells via diverse mechanisms [27]. MDSCs suppress antitumor immunity by releasing immunoregulatory molecules such as arginase (ARG), inducible nitric oxide synthase (iNOS), TGF-β, and IL-10. These factors impair T cell proliferation and diminish tumor-specific T cell activity. Additionally, like TAMs, MDSCs secrete galectin-9, which interacts with T-cell immunoglobulin and mucin-domain containing-3 (TIM-3) on T cells, inducing apoptosis [28]. MDSCs inhibit the cytotoxicity and cytokine release of NK cells (e.g., interferon-γ (IFN-γ)), which is mediated by the NKp30 receptor [29]. MDSCs contribute to immunosuppression by facilitating the expansion of CD4^+^CD25^+^Foxp3^+^ Tregs. Additionally, in advanced HCC, MDSCs have been shown to interact with Kupffer cells, resulting in increased PD-L1 expression within the TME. The pivotal role of MDSCs has prompted researchers to explore targeted therapeutic strategies against these cells. Trabectedin, a novel chemotherapeutic agent, selectively targets tumor cells and induces apoptosis in bone marrow cells [30]. In a clinical study, it significantly reduced disease progression and mortality rates compared to best supportive care in patients with advanced malignancies [31]. Trabectedin has been shown to decrease intratumoral MDSC levels, potentially by downregulating MDSC-promoting factors like G-CSF secreted by TAFs [32]. In addition to reducing MDSC-promoting factors, Trabectedin can directly trigger MDSC apoptosis. By lowering MDSC abundance and modulating their suppressive activity, it enhances antitumor immune recognition and response [33]. Estrogen has been reported to impair the functional activity of myeloid cells in HCC [34]. Estrogen lowers the risk of liver cancer in female mice by suppressing Janus kinase activation and inhibiting the STAT6 signaling pathway in necrotic tumor cells. Epidemiological data also show that HCC incidence is generally higher in men than in women, particularly among older populations [35]. However, female HCC patients often have a better survival rate [36]. Estrogen can promote the activity of T cells and NK cells, and also enhance the immune recognition and elimination of tumor cells by modulating the function of DCs [37]. In female patients with HCC, estrogen may confer protection by promoting cellular immune activity and suppressing immune tolerance. Further studies are needed to clarify the regulatory mechanisms of MDSCs and their potential as therapeutic targets, which could provide new directions for liver cancer treatment strategies.

### 2.3 Regulatory T Cells (Tregs)

The TME in HCC is characterized by substantial lymphocytic infiltration, including B cells, tertiary lymphoid structures, and various T cell populations collectively known as tumor-infiltrating lymphocytes (TILs). TILs modulate both innate and adaptive immune responses, thereby influencing tumor progression. Among these, multiple T cell subsets—such as αβ T cells, γδ T cells, CD4^+^ T cells, and CD8^+^ T cells—play central roles in orchestrating immune dynamics within the TME [38]. Evidence indicates that various T lymphocyte subsets fulfill distinct functions within the tumor microenvironment. Among them, CD4^+^ Tregs suppress antitumor immunity by inhibiting the activity of effector T cells. Tregs serve a dual role: they help maintain immune homeostasis and prevent autoimmunity, while also contributing to transplant tolerance. Although Treg accumulation in inflamed or tumor-infiltrated liver tissue may protect against collateral damage to normal hepatocytes, their immunosuppressive activity fosters immune tolerance toward tumor cells. This ultimately impairs the cytotoxic function of tumor-specific effector cells, such as CD8^+^ cytotoxic T lymphocytes (CTLs), within the TME [39]. In HCC, the presence of Tregs within tumors and peripheral blood appears more influential than that of CD8^+^ T cells. Studies have reported a marked elevation in both the frequency and absolute number of CD4^+^CD25^+^ Tregs in the TME, along with higher peripheral Treg levels in HCC patients compared to individuals with HCV infection or healthy controls. Tregs mediate immunosuppression through multiple mechanisms, including the release of inhibitory cytokines, induction of cytolysis, metabolic disruption, and suppression of dendritic cell function via CTLA-4 and indoleamine 2,3-dioxygenase (IDO). Notably, the secretion of TGF-β and IL-10 by Tregs within the TME impairs CD8^+^ T cell activity [40]. The collaboration between Tregs and Kupffer cells results in the release of IL-10, STAT3, and VEGF, which triggers Treg proliferation while inhibiting KCs from producing CD8^+^ T cells. It has been discovered that liver-specific chemokine receptor CCR6 draws Tregs into tumors where they are attracted by HCC cell-released CCL20. Additionally, various immune characteristics have been identified in Tregs including PD-1, CTLA-4, OX40, GITR, and TIM-3 [41]. These markers have been evaluated in both preclinical and clinical studies to assess their roles in chronic inflammation and tumor progression. Immunological analyses have revealed that Tregs can effectively suppress CD8^+^ T cell function. Spatial distribution studies indicate that Tregs are more concentrated in the tumor core, whereas CD8^+^ T cells are primarily localized at the tumor margins.

### 2.4 Dendritic Cells (DCs)

DCs, key antigen-presenting cells (APCs), are essential for antigen presentation, regulation of T cell differentiation, and modulation of T cell responses. In the human liver, DCs are primarily classified into plasmacytoid DCs (pDCs) and classical DCs (cDCs). The pDCs, characterized by BDCA-2 and CD123 expression, respond to TLR7/8 ligands and contribute to antiviral defense through type I interferon secretion, though their capacity to activate T cells is limited. The cDCs, also known as myeloid DCs, are subdivided into CD141^+^/CD14^−^ cDC1 and CD1c^+^/CD14^+^ cDC2 subsets. Both cDC1 and pDCs activate CD4^+^ T cells, while cDC1 and cDC2 facilitate MHC I–restricted activation of CD8^+^ T cells, supporting effector and memory T cell responses. Notably, cDC2 are abundant in the human liver but scarce in the spleen [42]. They contribute to antiviral immunity through type I interferon secretion but exhibit limited capacity to activate T cells [43]. Tumor cells promote the differentiation of DCs into an immature state by downregulating antigen presentation and adhesion molecules, while secreting immunosuppressive factors like IL-10 and VEGF. These immature DCs, in turn, activate Tregs, fostering immune tolerance and suppressing the activity of effector T cells [44]. Studies suggest a potential interaction between immunosuppressive Tregs and pDCs in HCC. DC vaccines are under investigation as a therapeutic approach, wherein patients are administered activated, mature DCs to elicit antitumor immune responses [45]. The study demonstrated favorable safety and tolerability profiles, suggesting that combining DC vaccines with immune checkpoint inhibitors could be a promising strategy for treating malignancies.

### 2.5 Kupffer Cells (KCs)

KCs, as a specialized subset of macrophages located within the hepatic sinusoids, constitute an integral component of the innate immune system. They originate from monocytes that adhere to the liver sinusoidal walls and undergo differentiation. Under specific physiological circumstances, phagocytosis serves as a rapid mechanism for eliminating exogenous particles or erythrocytes from the bloodstream [46]. In recent years, an increasing number of studies related to KCs have shown their significant impact on the early diagnosis and treatment of HCC [47]. KCs in the pathological state of TME can promote the activation of hepatic stellate cells, thereby promoting fibrosis of liver tissue, producing extracellular matrix (ECM), and triggering tumors. KCs are a key mediator for inflammatory reactions in liver tissue cells, and M2-type KCs express high levels of TGF-β1. Activate hepatic stellate cells (HSCs), which secrete ECM and promote liver fibrosis. When the body is in a normal state, there is always a dynamic balance between the production and degradation of ECM. During liver fibrosis, this balance is disrupted, causing ECM to accumulate and the liver to transform from normal to liver fibrosis or liver cancer. Meanwhile, the excessive accumulation of ECM may also cause local hypoxia, create an inflammatory microenvironment, and reduce the likelihood of the body effectively recognizing and killing liver cancer cells [48]. Additionally, Kupffer cells express α 1-Adrenergic Receptors (α 1-ARs) which can facilitate tumor initiation and metastasis through sympathetic nervous system modulation. Liver cancer is more prevalent in cases of liver cirrhosis following chronic inflammation, and in advanced stages of liver cirrhosis, the sympathetic nervous system (SNS) exhibits abnormal hyperactivity, with SNS innervation playing a crucial role in HCC development. Studies have demonstrated that increased density of sympathetic nerve fibers and KCs α 1-ARs are associated with unfavorable prognosis in HCC; interventions such as sympathetic denervation or α 1-AR blockade have shown potential to reduce diethyl nitrosamine-induced liver cancer incidence and progression. Further investigations revealed that SNS activation via α 1-ARs promotes Kupffer cell activation, and sustains an inflammatory microenvironment, thereby facilitating HCC occurrence [49].

### 2.6 Liver Sinusoidal Endothelial Cells (LSECs)

LSECs are specialized endothelial cells that form the interface between circulating blood cells, hepatocytes, and hepatic stellate cells. Under physiological conditions, LSECs modulate hepatic vascular tone to maintain low portal pressure and stable blood flow. They also help preserve the quiescent state of hepatic stellate cells, thereby preventing intrahepatic vasoconstriction and fibrogenesis. Under pathological conditions, especially in HCC, LSECs significantly contribute to disease initiation and progression [50]. From a pathological perspective, it is believed that HCC progresses from precancerous lesions (low to high-grade developmental nodules) to early and late-stage HCC, originating from highly vascularized tumors. This process is closely linked to endothelial cell remodeling and contributes to both HCC development and metastasis. Tumor-driven capillarization of LSECs promotes HSC activation and fibrogenesis, whereas maintenance of the non-capillarized (fenestrated) phenotype helps inhibit HSC activation and protect against liver injury. Chemokines such as CXCL9 and CXCL16 regulate immune cell infiltration during liver disease progression, including in HCC. Furthermore, increased adhesion between circulating tumor cells and LSECs, along with LSEC-induced immune tolerance within the tumor microenvironment, facilitates metastatic dissemination of HCC cells [51]. As HCC progresses, LSECs in peritumoral regions undergo phenotypic alterations, including reduced expression of characteristic markers such as stabilin-2 and CD32b. In mouse xenograft models, peritumoral liver tissue exhibits increased microvascular density and elevated expression of proangiogenic genes, including IL-6 and IL-6R, compared to tumor core tissue [52]. Peritumoral endothelial cells isolated from HCC patients show greater proliferative capacity in response to IL-6 and soluble IL-6R stimulation compared to endothelial cells from tumor tissue. These findings highlight the active involvement of peritumoral endothelial cells in HCC progression and emphasize their relevance in exploring tumor–microenvironment interactions.

### 2.7 Hepatic Stellate Cells (HSCs)

HSCs, residing in the space of Disse, are pericyte-like cells that account for approximately 5%–10% of the total liver cell population [53,54]. HSCs participate in liver regeneration but exhibit inhibitory effects on liver cancer by secreting cytokines such as hepatocyte growth factor (HGF) and IL-6 [55]. Cirrhosis, as the main cause of HCC, is caused by long-term chronic inflammation leading to sustained activation of HSCs, ultimately leading to excessive scar formation in the liver, which in turn develops into fibrosis and cirrhosis, ultimately leading to HCC [56]. Several components of the TME, including TGF-β, platelet-derived growth factor (PDGF), as well as vasoactive agents such as thrombin, angiotensin, and endothelin-1, contribute to the activation of HSCs [57]. The activation of HSCs also leads to changes in myofibroblast-like phenotype, production of ECM proteins, and infiltration of HCC. It can also lead to the activation of hematopoietic stem cells, promote the development of accumulated TME, and further maintain the stability of HCC cells [58]. In the process of HCC metastasis, the regulation of immune cells plays a crucial role in tumor immune evasion. The activation of HSCs can affect immune cells and promote their entry into an immunosuppressive state. Research has shown that the transdifferentiation of HSCs increases the production and content of extracellular vesicles (EVs). When modified EVs reach the macrophage membrane, they stimulate cytokine synthesis and release, as well as macrophage migration [59]. Filliol et al., demonstrated that in early disease stages, minimally activated or quiescent HSCs secrete hepatocyte growth factor (HGF), which suppresses HCC progression. However, in more advanced stages, HSCs become fully activated and begin producing collagen I, thereby facilitating tumor growth [60]. The regulatory signals driving the functional heterogeneity of HSC subpopulations remain largely unexplored. Moreover, during fibrosis resolution, activated HSCs may either undergo apoptosis or transition into an inactivated state, which differs from their original quiescent form [61]. The involvement of HSCs in tumor metastasis is primarily mediated through their activation, which promotes EMT in HCC cells, facilitating metastatic spread. Elucidating how activated HSCs contribute at various stages of tumor development is essential for a deeper understanding of HCC pathogenesis.

### 2.8 Cancer-Associated Fibroblasts (CAFs)

CAFs are integral components of the tumor stroma and modulate cancer progression through diverse mechanisms, including the secretion of growth factors, inflammatory mediators, exosomes, and the regulation of ECMn remodeling, angiogenesis, tumor biology, and therapeutic response [62,63]. Since most HCC arises in the context of liver cirrhosis, which involves fibroblast activation, proliferation, and accumulation, CAFs contribute to a microenvironment conducive to tumor initiation, progression, and metastasis [64]. During HCC development, tumor cells release various factors that recruit CAFs into TME. In turn, CAFs secrete a range of soluble molecules—including growth factors, inflammatory cytokines, chemokines, and angiogenic factors—that promote tumor cell proliferation and dissemination [65]. Their crosstalk with cancer cells is mediated through a complex signal network that includes TGF-β, mitogen-activated protein kinase (MAPK), Wnt/β-signal pathways such as catenin, Janus kinase/signal transduction, and transcription activating factors (JAK/STATs), and epidermal growth factor receptors (EGFR) [66]. In recent studies on the role of CAFs in the progression of HCC, it was found that CAFs are involved in various chemokines in the TME endocrine system by activating Hedgehog or TGF-β pathways to promote the invasion and metastasis of HCC cells [67]. The TGF-β signaling pathway exhibits dual functionality—acting as a tumor suppressor in precancerous cells while promoting tumor progression in malignant cells. In the TME, TGF-β/Smad signaling facilitates EMT in tumor endothelial cells by upregulating Snail and Slug, thereby enhancing angiogenesis and the accumulation of myofibroblasts and CAFs. Emerging evidence also highlights the broad involvement of the JAK/STAT pathway, activated by CAFs, in regulating key oncogenic processes such as cellular plasticity, proliferation, migration, EMT, angiogenesis, and metastasis. In HCC, CAF-derived IL-6 triggers EMT in cancer cells via activation of the IL-6/JAK/STAT3 axis, which induces transglutaminase 2 (TG2) expression and promotes the mesenchymal phenotype [68]. Numerous studies emphasize the importance of interactions between HCC cells, CAFs, and other stromal components in driving tumor progression. While preclinical and clinical evidence has begun to uncover the involvement of CAFs in immune evasion and resistance to immunotherapy, further elucidation of their distinct roles in HCC could support the development of more effective molecularly targeted therapies.

### 2.9 Cancer Stem Cells (CSCs)

CSCs represent a subpopulation within tumors that contribute to invasion, metastasis, recurrence, and resistance to therapy. They interact with various cytokines in TME, jointly promoting HCC progression. IL-6 secreted by TAMs in HCC activates STAT3 signaling, thereby enhancing the proliferation of CSCs. Additionally, IL-17E produced by non-CSCs promotes CSC proliferation and self-renewal through activation of the JAK/STAT3 and NF-κB signaling pathways in HCC [69]. Moreover, STAT3 enhances the expression of matrix metalloproteinases (MMP-2 and MMP-9), which contribute to ECM degradation and facilitate the upregulation of EMT-related transcription factors, such as Slug and Twist, ultimately promoting the invasive potential of CSCs in HCC [70]. CAFs secrete cytokines, chemokines, and growth factors to improve the self-renewal of CSCs. IL-6 and HGF produced by CAFs activate STAT3 signaling, leading to CSC activity [71]. The CSCs model for HCC suggests that tumor growth is driven by a subset of tumor stem cells in cancer. This model helps to explain several clinical features of HCC, such as the frequent recurrence following initially successful chemotherapy or radiotherapy, as well as tumor dormancy and resistance to treatment. Over the past two decades, growing research efforts have focused on the identification and characterization of liver CSCs, paving the way for the development of novel diagnostic tools and therapeutic strategies for HCC [72].

## 3 Immune Checkpoint Inhibitors (ICIs)

**Figure 2:**
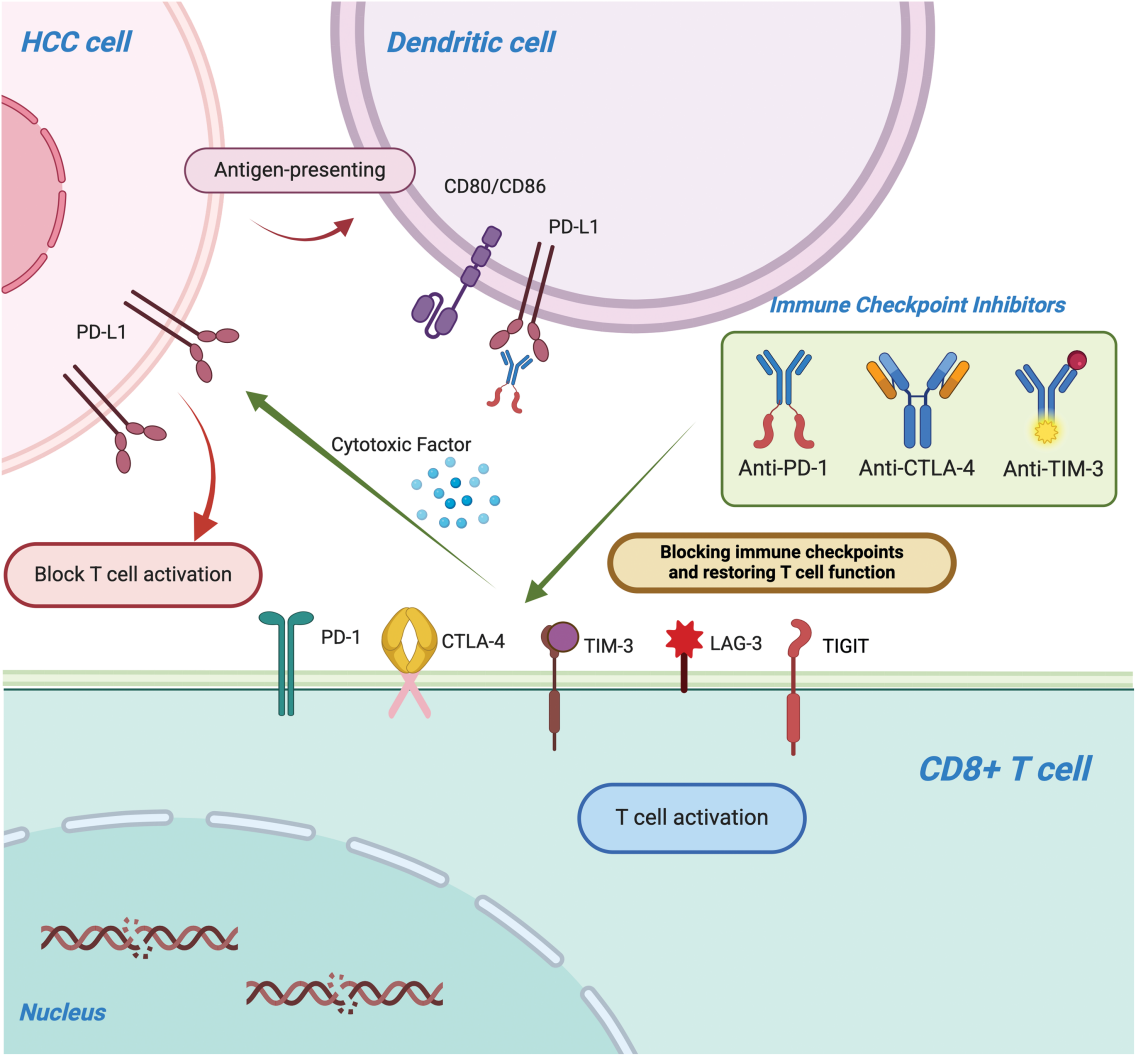
The figure illustrates the mechanism by which immune checkpoint molecules, including PD-1/PD-L1, CTLA-4, TIM-3, lymphocyte activation gene-3 (LAG-3), and T cell immunoreceptor with Ig and ITIM domains (TIGIT), suppress T-cell function and enable tumors to evade immune surveillance. Immune checkpoint inhibitors (ICIs), such as anti-PD-1, anti-PD-L1, and anti-CTLA-4 monoclonal antibodies, restore T-cell activity by blocking these inhibitory pathways

Immune checkpoints are a class of regulatory molecules within the immune system that modulate the activity of immune cells, maintaining the balance of immune responses and preventing excessive immune activation that could lead to autoimmune diseases or tissue damage [73]. These checkpoint molecules primarily function by interacting with receptors and ligands on immune cell surfaces, either inhibiting or promoting immune cell activation and function, thus enabling negative regulation of immune responses. For example, PD-1 (programmed cell death protein 1) and its ligand PD-L1 are widely studied immune checkpoints; their interaction inhibits T-cell function, aiding tumor cells in evading immune surveillance [74]. Similarly, CTLA-4 (cytotoxic T-lymphocyte-associated protein 4) inhibits T-cell activation by binding to CD80/CD86, thereby limiting the intensity and duration of immune responses [75]. Under normal physiological conditions, immune checkpoints play a crucial role in immune tolerance, preventing the immune system from attacking self-tissues. However, tumor cells often exploit these checkpoints by upregulating their expression to escape immune detection, promoting tumor growth and metastasis [76]. In recent years, immune checkpoint inhibitors (such as anti-PD-1 and anti-CTLA-4 antibodies) have emerged as a novel immunotherapeutic approach. By blocking these immune checkpoints, these inhibitors restore immune system function, reactivate T-cells and other immune cells, and enhance the immune system’s ability to recognize and eliminate tumors, representing a significant breakthrough in cancer therapy (Fig. 2). Immune checkpoint inhibitors can lead to a spectrum of immune-related adverse events (irAEs), most commonly affecting the skin, gastrointestinal tract, liver, and endocrine organs. Prompt recognition and appropriate management—often involving corticosteroids or other immunosuppressive agents—are critical to mitigate severity and prevent long-term complications (Table 1).

**Table 1:** Common immune-related adverse events and management strategies

Organ system	Common irAEs	Management strategy
Skin	Rash, pruritus, vitiligo	Topical steroids, systemic steroids if severe
Gastrointestinal	Diarrhea, colitis	Oral or intravenous corticosteroids, infliximab
Liver	Immune-mediated hepatitis	Corticosteroids: mycophenolate mofetil if needed
Endocrine	Thyroiditis, hypophysitis, diabetes	Hormone replacement therapy, corticosteroids
Lung	Pneumonitis	High-dose corticosteroids, oxygen therapy

### 3.1 Anti-PD-1/Anti-PD-L1

The protein PD-1 can be expressed by various types of cells, including active CD8+ T cells, CD4+ T cells, B cells, Tregs, NKs, myeloid cells, monocytes, and DCs. During the effect phase of T cells, PD-1 primarily provides negative regulatory signals. The maintenance of immune tolerance and inhibition of cytotoxic T cells are of utmost importance [77]. PD-1 binds to either PD-L1 or PD-L2 to inhibit the immune system’s response against tumors [78]. PD-L1 is expressed in various non-immune cells as well as tumor cells, B cells, T cells, DCs, antigen-presenting cells (APCs), and MDSCs. In the TME of HCC, PD-L1 is predominantly found in tumor cells and some other APCs. On the other hand, PD-L2 expression is limited to APCs only, which may explain why anti-PD-1/PD-L1 antibodies are effective while PD-L2 has minimal impact on antitumor immunity. In TME, multiple factors interact to affect the expression of PD-1 and PD-L1. For example, Y-box binding protein 1 (YBX1) and circRNA can regulate the expression of PD-L1 and inhibit the function of effector T cells [79,80]. In HCC cases, ICIs can prevent the interaction between PD-1 and PD-L1 from inducing immunosuppression. Patients with high levels of PD-1 or PD-L1 expression in HCC tend to have a poorer prognosis [81]. Among them, several classical drugs have been widely used in clinical practice, including nivolumab, pembrolizumab, durvalumab and atezolizumab.

Nivolumab is a monoclonal antibody that acts as a PD-1 inhibitor, which was approved by the US FDA in June 2015. By obstructing the interaction between PD-1 and its ligands, namely PD-L1 and PD-L2, Nivolumab enhances the activation of T cells to recognize cancer cells, thereby facilitating their elimination [82]. Durvalumab is currently the most frequently utilized FDA-approved PD-L1 monoclonal antibody, and it exhibits a superior safety and tolerability profile compared to conventional chemotherapy drugs. In ongoing clinical trials, it has demonstrated its ability to effectively impede the proliferation of HCC cells, reduce tumor volume, and prolong overall survival. The Phase 2 trial investigated the combination of durvalumab with tremelimumab or bevacizumab in patients diagnosed with unresectable hepatocellular carcinoma (uHCC) [83]. The co-inhibition of PD-L1 by bevacizumab and VEGF inhibition may exhibit an additive effect, enhancing clinical activity [84]. The introduction of atezolizumab has significantly advanced the treatment of advanced unresectable HCC. The FDA has approved the first-line treatment of advanced hepatocellular carcinoma (aHCC) to atezolizumab and bevacizumab, based on the findings from the IMbrave 150 study [85].

### 3.2 Anti-CTLA-4

The CTLA-4 co-receptor functions as an inhibitor of immune responses and belongs to the CD28 co-receptor family. It is predominantly expressed in activated T cells, regulatory T cells, and naive T cells. In immature T cells, CTLA-4 resides intracellularly but translocates to the cell surface upon stimulation. In Tregs, it remains constitutively present and aids in suppressing immune responses. CTLA-4 competes with CD28 for binding to the costimulatory molecules CD80 (B7-1) and CD86 (B7-2) on antigen-presenting cells, such as DCs, with significantly higher affinity than CD28. While the interaction between B7-1 and CD28 promotes T cell activation, its engagement by CTLA-4 initiates inhibitory signaling pathways that suppress immune responses [86]. Monoclonal antibodies like ipilimumab and tremelimumab can block this negative regulatory response and are well tolerated. In 1996, Allison and his colleagues demonstrated through animal models that inhibitory antibodies that block CTLA-4 can boost the immune response against tumors, marking the first successful application of CTLA-4 in cancer treatment [87]. Tremelimumab is an inhibitor of CTLA-4 that belongs to the human IgG2 class. One of the earliest known practical immunotherapy experiments is to apply tremelimumab to patients with HCC and HCV-related cirrhosis who have progressed after treatment with sorafenib [88]. The utilization of these medications in isolation is infrequent, as they are commonly employed in conjunction with other therapies due to their distinctive mechanisms of action. The efficacy of tremelimumab, the anti-CTLA4, treatment in combination with durvalumab has been demonstrated in numerous phase I/II trials for advanced HCC.

### 3.3 Anti-TIM-3

TIM-3 (T-cell immunoglobulin and mucin-domain containing-3) is an immune checkpoint molecule predominantly expressed on immune cells such as effector T cells (Teff), Tregs, NK cells, and DCs [89]. Through interactions with its ligands, including galectin-9, carcinoembryonic antigen-related cell adhesion molecule 1 (CEACAM1), and phosphatidylserine, TIM-3 inhibits T-cell function, promotes immune tolerance, and ultimately facilitates tumor immune evasion. In HCC, high TIM-3 expression is considered a critical marker of T-cell exhaustion, which directly impairs anti-tumor immune responses [90]. Studies have demonstrated that TIM-3 is highly expressed in CD8+ T cells and Tregs within tumor tissues and peripheral blood of HCC patients. This expression often correlates with increased tumor burden, enhanced invasiveness, higher recurrence rates, and is closely associated with disease progression and poor prognosis [91]. As an immune checkpoint molecule, TIM-3 can be targeted with inhibitors to restore T-cell function and enhance anti-tumor immunity [92]. Monoclonal antibodies targeting TIM-3, such as MBG453 and TSR-022, have entered preliminary stages of clinical application, with evidence suggesting their potential therapeutic efficacy against HCC. Furthermore, a synergistic relationship exists between TIM-3 and the PD-1/PD-L1 pathway, with their co-expression often indicating a more profound state of T-cell exhaustion [93]. Preclinical and clinical studies in HCC have shown that dual blockade of TIM-3 and PD-1/PD-L1 enhances anti-tumor activity. This combination therapy improves the immunosuppressive TME and increases treatment response rates.

### 3.4 Anti-LAG-3

Lymphocyte-activation gene 3 (LAG-3) is a membrane protein predominantly expressed in immune cells such as CD4+ T cells, CD8+ T cells, Tregs, and NK cells. LAG-3 interacts with major histocompatibility complex class II (MHC-II) molecules to suppress T-cell proliferation, cytokine secretion, and cytotoxic function, thereby weakening anti-tumor immune responses [94]. It is frequently co-expressed with PD-1, collectively contributing to T-cell exhaustion. The high expression of LAG-3 on Tregs enhances their immunosuppressive capabilities, further promoting tumor immune evasion. LAG-3 inhibitors, such as Relatlimab, have demonstrated promising preclinical and clinical potential in various malignancies. Blocking LAG-3 restores T-cell function and bolsters anti-tumor immunity. In HCC, research is ongoing to evaluate the safety and efficacy of LAG-3 monoclonal antibodies. LAG-3 inhibitors have entered phase I/II clinical trials, including studies of Relatlimab in combination with Nivolumab for the treatment of HCC [95]. These trials aim to assess the safety, tolerability, and preliminary efficacy of the combination therapy. Early results indicate that dual blockade of LAG-3 and PD-1 exhibits significant anti-tumor activity with manageable toxicity profiles, highlighting its potential as a novel immunotherapy approach for HCC.

### 3.5 Anti-TIGIT

T-cell immunoglobulin and ITIM domain (TIGIT) is an immune checkpoint molecule whose expression is significantly upregulated in TME of HCC. TIGIT is primarily distributed on T cells, NK cells, and other immunosuppressive cell populations. By binding to its ligand CD155, TIGIT inhibits the activity of effector T cells and NK cells while enhancing the function of Tregs, thereby establishing an immunosuppressive TME [96]. This mechanism not only facilitates immune evasion by tumor cells but is also closely associated with HCC progression and poor prognosis. TIGIT monoclonal antibodies, as immune checkpoint inhibitors, have advanced to clinical trial stages. Blocking TIGIT alone or in combination with PD-1/PD-L1 antibodies can restore T-cell and NK cell function, strengthening anti-tumor immune responses. Furthermore, TIGIT inhibitors can be integrated with existing therapies such as targeted agents (e.g., sorafenib and lenvatinib) or locoregional treatments (e.g., ablation and embolization) to improve the TME and enhance therapeutic sensitivity [97]. For HCC patients, combination therapies may overcome the limitations of single-agent approaches, thereby improving overall treatment outcomes.

## 4 Combination Therapy

Given the heterogeneity of HCC and the complexity of the tumor microenvironment, a single therapeutic approach frequently falls short of addressing clinical requirements. Consequently, combination therapy strategies, such as the integration of ICI with immunotherapy, ICI with targeted therapies, chemotherapy, and radiotherapy, have emerged as pivotal research directions in HCC treatment. The following highlights some of the most recent advancements in clinical research. In the table below, we present detailed information on clinical trials of ICI therapy that further substantiate the potential and efficacy of immunotherapy (Table 2).

**Table 2:** Clinical trials evaluating the efficacy of immunotherapy in conjunction with other therapeutic modalities or pharmacological agents

NCT number	Interventions	Drug combinations	Phase	Status	Primary outcome measures	Result
NCT03298451	Durvalumab + Tremelimumab	Anti-PD-1 + anti-CTLA-4	III	Completed	OS	36-month OS rate: 30.7% 48-month OS rate: 25.2%
NCT03092895	SHR-1210	Anti-PD-1	II	Completed	OS; PFS	mPFS: 3.7 months mOS: 13.2 months
NCT04072679	Sintilimab + IB1305	Anti-PD-1 + anti-VEGF	Ib	Completed	OS; PFS	ORR: 34%(mRECIST) DCR: 78% mPFS: 10.5 months mOS: 20.2 months
NCT03006926	Pembrolizumab + Lenvatinib	Anti-PD-1 + TKI	Ib	Completed	OS; PFS	mPFS: 9.3 months mOS: 22 months
NCT04042805	Sintilimab + Lenvatinib	Anti-PD-1 + TKI	II	Completed	ORR; OS; PFS	ORR: 36.1% (mRECIST) DCR: 94.4% mPFS: 14.3 months
NCT03434379	Atezolizumab + Bevacizumab	Anti-PD-1 + anti-VEGF	III	Completed	OS; PFS	12-month OS rate: 67.2% mPFS: 6.8 months
NCT03713593	Pembrolizumab + Cabozantinib	Anti-PD-1 + TKI	III	Completed	OS; PFS	ORR: 40.8% (mRECIST) mPFS: 8.2 months mOS: 21.2 months
NCT03764293	Camrelizumab + Apatinib	Anti-PD-1 + anti-VEGFR2/TKI	III	Completed	OS; PFS	mPFS: 5.6 months mOS: 22.1 months
NCT03970616	Durvalumab + Tivozanib	Anti-PD-1 + anti-VEGFR1-3/TKI	Ib/II	Completed	OS; PFS	2 of 7 achieving PR
NCT03781960	Nivolumab + Abemaciclib	Anti-PD-1 + anti-CDK4/6	II	Completed	OS; PFS	/
NCT03519997	Pembrolizumab + Bavituximab	Anti-PD-1 + anti-PS	II	Completed	OS; PFS	mPFS: 6.3 months
NCT04172571	AK105 + Anlotinib	Anti-PD-1 + TKI	Ib/II	Completed	ORR; OS; PFS	ORR: 31% (mRECIST) DCR: 82.8% mPFS: 8.8 months 12-month OS rate: 69%
NCT03753659	Pembrolizumab + Radiofrequency ablation	Anti-PD-1+ RFA	II	Completed	ORR	ORR: 13.3%(RECIST1.1)
NCT04770896	Atezolizumab + Sorafenib	Anti-PD-1 + TKI	III	Recruiting	OS; PFS	/
NCT05199285	Candonilimab + Bevacizuma	Anti-PD-1/CTLA-4 + anti-VEGF	II	Recruiting	Safety; efficacy	/
NCT04522986	HLX53 + Serplulimab + Bevacizuma	Anti-TIGIT + Anti-PD-1+ anti-VEGF	II	Recruiting	Safety; efficacy	/
NCT04605731	Durvalumab + Tremelimumab + Y-90	Anti-PD-L1 + anti-CTLA-4 + TARE	II	Recruiting	pCR	/
NCT04829383	Atezolizumab + Bevacizumab	Anti-PD-1 + anti-VEGF	II	Recruiting	OS; PFS	/
NCT05134532	Atezolizumab + Bevacizumab + Regorafenib	Anti-PD-1 + anti-VEGF + TKI	II	Recruiting	Safety; efficacy	/
NCT04039607	Nivolumab + Ipilimumab	Anti-PD-1 + anti-CTLA-4	III	Recruiting	OS; PFS	/
NCT05665348	Atezolizumab + Bevacizumab + Ipilimumab	Anti-PD-1 + anti-VEGF + anti-CTLA-4	III	Recruiting	OS; PFS	/

Note: PD-1: Programmed Cell Death Protein 1; PD-L1: Programmed Death-Ligand 1; CTLA-4: Cytotoxic T-Lymphocyte Antigen 4; VEGF: Vascular Endothelial Growth Factor; VEGFR: Vascular Endothelial Growth Factor Receptor; TKI: Tyrosine Kinase Inhibitor; CDK4/6: Cyclin-Dependent Kinases 4 and 6; PS: Phosphatidylserine; TIGIT: T-cell Immunoglobulin and ITIM Domain; RFA: Radiofrequency Ablation; OS: Overall Survival; PFS: Progression-Free Survival; ORR: Objective Response Rate.

In patients with advanced HCC, the efficacy of single-agent ICIs is often limited. Combination ICI therapies have introduced new avenues for treatment. The concurrent use of PD-1/PD-L1 inhibitors (e.g., nivolumab, pembrolizumab) and CTLA-4 inhibitors (e.g., ipilimumab) has demonstrated promising clinical outcomes across various tumor types. For HCC, studies indicate that combining PD-1/PD-L1 and CTLA-4 inhibitors can significantly enhance T-cell activation and restore anti-tumor immune responses, particularly in patients who exhibit poor responses to monotherapy [77]. In the KEYNOTE-524 study, the combination of pembrolizumab (PD-1 inhibitor) and ipilimumab (CTLA-4 inhibitor) demonstrated superior efficacy compared to monotherapy [98]. Additionally, the combination of ICIs with immune stimulators, such as anti-CD40 or anti-OX40 antibodies, further amplifies the immune system’s ability to recognize and eliminate tumors [99]. Tyrosine kinase inhibitors (TKIs), as a key class of targeted therapies, play a pivotal role in the treatment of HCC by precisely disrupting aberrant signaling pathways involved in tumor growth and angiogenesis (Table 3). Combining ICIs with targeted therapies not only restores immune responses but also precisely inhibits tumor growth, emerging as a widely adopted and well-established clinical strategy. Common combinations include PD-1/PD-L1 inhibitors with VEGF inhibitors or multi-targeted therapies. In the KORONA study, the combination of nivolumab (PD-1 inhibitor) and bevacizumab (VEGF inhibitor) demonstrated higher overall response rates and longer survival in patients with advanced HCC [100]. Similarly, recent studies have demonstrated that the combination of nivolumab and lenvatinib exhibits high clinical efficacy and tolerability, presenting a promising treatment option for advanced HCC [101]. Although chemotherapy has not shown strong advantages in HCC treatment strategies, its role has evolved with the advent of ICIs and interventional therapies. Hepatic arterial infusion chemotherapy (HAIC) combined with ICIs has emerged as an effective neoadjuvant therapy for HCC [102]. Chemotherapy can reduce immunosuppressive cell populations within the tumor microenvironment (e.g., Tregs and MDSCs) and suppress immunoinhibitory signals, thereby creating a favorable condition for ICI therapy. Simultaneously, ICIs relieve T-cell immune suppression, and when combined with the direct cytotoxic effects of chemotherapy, they further enhance the immune system’s anti-tumor efficacy.

**Table 3:** Current and future directions of combination strategies based on TKI plus ICIs in HCC treatment

Combination strategy	Agents involved	Clinical trial	Phase/year	Key results
Anti-VEGF + PD-L1 blockade	Atezolizumab + Bevacizumab	NCT03434379 (IMbrave150)	Phase III/2020	Median OS 19.2 mo; PFS 6.8 mo; ORR 27%
Multi-target TKI + PD-1 inhibitor	Lenvatinib + Pembrolizumab	NCT03713593 (LEAP-002)	Phase III/2023	Median OS 21.2 mo vs. 19.0 mo (NS); ORR 26%
MET/VEGFR2 inhibitor + PD-L1 blockade	Cabozantinib + Atezolizumab	NCT03755791 (COSMIC-312)	Phase III/2022	PFS improved (6.8 vs. 4.2 mo); OS not significantly improved
FGFR4 inhibitor + PD-1 inhibitor	FGF401 + spartalizumab	NCT02325739	Phase I/2020	Early safety data promising; PFS data pending
Anti-VEGF/PD-1 dual blockade	Apatinib + SHR-1210 (camrelizumab)	NCT02989922	Phase II/2021	ORR 34.3%; manageable toxicity
Novel TKI + PD-1 blockade	Donafenib + sintilimab	NCT04980467	Phase II/ongoing	Early reports show good tolerability

## 5 Bispecific Antibody

Bispecific antibodies (BsAbs) are engineered immunoglobulin constructs capable of simultaneously recognizing and binding to two distinct antigens or epitopes. Compared to conventional monospecific antibodies, BsAbs exhibit unique structural and functional advantages, enabling highly specific dual-targeted therapeutic strategies, particularly in oncology. The primary mechanism of action for BsAbs involves their ability to concurrently engage tumor-associated antigens (TAAs) and immune effector cells, effectively forming an immunological synapse that facilitates direct cytotoxic activity against tumor cells [103]. In HCC, BsAbs are designed to target overexpressed TAAs, such as Glypican-3 and alpha-fetoprotein (AFP), to achieve tumor-specific recognition [12]. Concurrently, they interact with immune effector cell receptors, including CD3 on T cells or CD16 on NK cells, promoting direct tumor-immune cell interactions and triggering robust anti-tumor immune responses. Additionally, BsAbs can simultaneously inhibit multiple oncogenic pathways. For example, VEGF/Ang-2 BsAbs effectively block key mediators of angiogenesis, thereby enhancing the efficacy of anti-angiogenic therapies in HCC [104]. Current investigations into Glypican-3, a highly specific antigen in HCC, have demonstrated that BsAbs targeting both Glypican-3 and CD3 can significantly enhance cytotoxic immune responses against tumor cells [105]. Advances in antibody engineering have driven the development of increasingly sophisticated BsAb platforms, including tri-specific antibodies and nanobody-based constructs, offering improved pharmacokinetics and tumor penetration. Looking forward, BsAbs are anticipated to play a pivotal role in the treatment of solid tumors, including HCC, and their integration with emerging immunotherapeutic modalities holds promise for overcoming therapeutic resistance and addressing the unmet clinical needs of patients with refractory or recurrent malignancies.

## 6 Conclusion

The TME plays a central role in the progression, immune evasion, and therapeutic resistance of HCC, making it both a major challenge and a promising target for treatment. Advances in immunotherapy, particularly ICIs, have offered a transformative approach to HCC management by reactivating anti-tumor immunity. However, the immunosuppressive components of the TME, including tumor-associated macrophages, Tregs, and MDSCs, limit the efficacy of ICIs when used alone. Combination therapies have emerged as a promising strategy to overcome these barriers. Dual checkpoint blockade, such as the combination of PD-1/PD-L1 and CTLA-4 inhibitors, has shown the potential to synergistically activate T cells and restore immune function. Additionally, integrating ICIs with anti-angiogenic agents, multi-kinase inhibitors, or TME-targeting approaches has demonstrated the ability to modulate the TME, enhance immune cell infiltration, and inhibit tumor progression. These approaches not only address the heterogeneity of HCC but also provide opportunities to reprogram the immune landscape of the tumor. Despite the encouraging progress, challenges such as immune-related toxicities, therapeutic resistance, and patient-specific variations in TME composition remain significant hurdles. Addressing these issues will require a deeper understanding of the molecular and cellular dynamics within the TME. Developing predictive biomarkers, tailoring combination regimens, and optimizing treatment sequencing are critical steps to refine these therapeutic strategies. In the future, leveraging insights into the TME to guide personalized immunotherapy approaches holds the potential to revolutionize HCC treatment. By targeting the complex interactions within the TME, combination therapies can achieve sustained anti-tumor responses, improve survival outcomes, and offer new hope for patients with this aggressive malignancy. As research continues to unravel the intricacies of the TME, immunotherapy will likely play an increasingly central role in transforming the landscape of HCC management.

## Data Availability

The datasets used and analyzed during the current study are available from the corresponding authors on reasonable request.
